# Fluent Speakers of a Second Language Process Graspable Nouns Expressed in L2 Like in Their Native Language

**DOI:** 10.3389/fpsyg.2017.01306

**Published:** 2017-08-03

**Authors:** Giovanni Buccino, Barbara F. Marino, Chiara Bulgarelli, Marco Mezzadri

**Affiliations:** ^1^Dipartimento di Scienze Mediche e Chirurgiche, Università Magna Graecia Catanzaro, Italy; ^2^Dipartimento di Psicologia, Università degli Studi di Milano-Bicocca Milan, Italy; ^3^Dipartimento di Discipline Umanistiche, Sociali e delle Imprese Culturali, Università degli Studi di Parma Parma, Italy

**Keywords:** embodied cognition, second language, semantics, objects, nouns

## Abstract

According to embodied cognition, language processing relies on the same neural structures involved when individuals experience the content of language material. If so, processing nouns expressing a motor content presented in a second language should modulate the motor system as if presented in the mother tongue. We tested this hypothesis using a go-no go paradigm. Stimuli included English nouns and pictures depicting either graspable or non-graspable objects. Pseudo-words and scrambled images served as controls. Italian participants, fluent speakers of English as a second language, had to respond when the stimulus was sensitive and refrain from responding when it was not. As foreseen by embodiment, motor responses were selectively modulated by graspable items (images or nouns) as in a previous experiment where nouns in the same category were presented in the native language.

## Introduction

Embodied cognition maintains that language processing involves the recruitment of the same sensory, motor, and even emotional neural substrates recruited when one executes, perceives or feels the content of language material (Glenberg, 1997; Barsalou, 1999; Pulvermüller, 2001; Gallese and Lakoff, 2005; Zwaan and Taylor, 2006; Jirak et al., 2010; Buccino et al., 2016). If so, then processing graspable objects and the corresponding nouns should modulate in a similar manner the motor system. Observing objects and manipulating them recruit a sensorimotor circuit, including premotor and parietal areas (Jeannerod et al., 1995; Binkofski et al., 1999; Chao and Martin, 2000; Grezes et al., 2003a,b). This circuit on one side codes for the intrinsic features of objects that make them appropriate for manual action; on the other it selects and implements the most appropriate actions to manipulate those objects. There is evidence that the recruitment of the motor system during object observation is finely tuned to the intrinsic features of objects (Buccino et al., 2009; Makris et al., 2011).

As for nouns, several studies showed a modulation of the motor system activity depending on the intrinsic features (e.g., size, type of prehension required to interact with them) of objects expressed by nouns (Glover et al., 2004; Tucker and Ellis, 2004; Lindemann et al., 2006; Myung et al., 2006; Bub et al., 2008; Cattaneo et al., 2010; Gough et al., 2012, 2013). Moreover, a specific modulation of hand motor responses has been shown during the processing of nouns referring to hand-related objects (Marino et al., 2013), as compared to foot-related objects. Two recent studies (Marino et al., 2014; Zhang et al., 2016) showed a similar modulation of the motor system during the processing of objects and nouns belonging to the same category. Additionally in an fMRI study (Desai et al., 2016), during reading nouns expressing graspable objects activations were found in areas also involved in action performance, thus supporting a grounded view of semantics. Taken as a whole, current literature supports the notion that processing visually presented graspable objects and nouns referring to the same object category recruit common neural substrates crucially involving the motor system (Ganis et al., 1996; Vandenberghe et al., 1996). In this context it is worth reminding that pivotal neurophysiological studies (for review see Pulvermüller et al., 2009) showed an early recruitment (within 200 ms from stimulus presentation) of the motor system during language processing. Furthermore in behavioral studies the modulation of the motor system during language processing may change over time moving from an early interference (operating between 100 and 200 ms after stimulus onset) to a later facilitation (operating when responses are requested later than 200 ms from stimulus presentation), as maintained by some models (see Chersi et al., 2010; Garcia and Ibanez, 2016). What about the processing of nouns expressing natural graspable objects when presented in a second language (L2)? If language is embodied and grounded in the sensory, motor and even emotional representations of the speaker coding for the language content, then during processing graspable nouns in fluent speakers of L2 we should find a modulation of the motor system similar to that found for nouns presented in their mother tongue (L1). In the present study, we assessed the modulation of motor responses in native Italian speakers with a high competence in English as L2 (Level C1 of the Common European Framework of Reference for Languages, CEFR), using the same paradigm of a previous study (Marino et al., 2014) where verbal stimuli were presented in L1. In that study participants were requested to give a semantic judgment, namely whether the presented stimulus was sensitive or meaningless, pressing a button at 150 ms after the stimulus onset. Native Italian speakers showed a specific modulation of hand motor responses (namely slower motor responses) during the processing of graspable items (presented as either pictures or nouns) as compared to non-graspable ones. We interpreted these results as a manifestation of the motor system being engaged in two tasks (processing the graspable picture or word and giving the hand motor response), thereby supporting the notion that the motor system is necessary to process language material expressing a motor content. The experimental hypothesis underlying the present study was that in a similar group of participants fluent speakers of English as L2, this task would lead to similar modulation of motor responses found during the presentation of comparable stimuli in the L1, as foreseen by embodiment.

## Methods

### Participants

Twenty-six right-handed undergraduate students from the University of Parma took part in the study (20 females; mean age = 22.07 ± 1.76 years). They were native Italian speakers who had an English language proficiency at the reference level C1 on the CEFR scale (Common European Framework of Reference for Languages: Learning, Teaching, Assessment). All had normal or corrected-to-normal vision, and reported no history of language disorders. They were unaware of the purpose of the experiment and gave their informed consent before testing. The study was conducted in accordance with the Declaration of Helsinki (1964) and the procedure recommended by the Italian Association of Psychology (AIP).

### Stimuli

**Figure 1:**
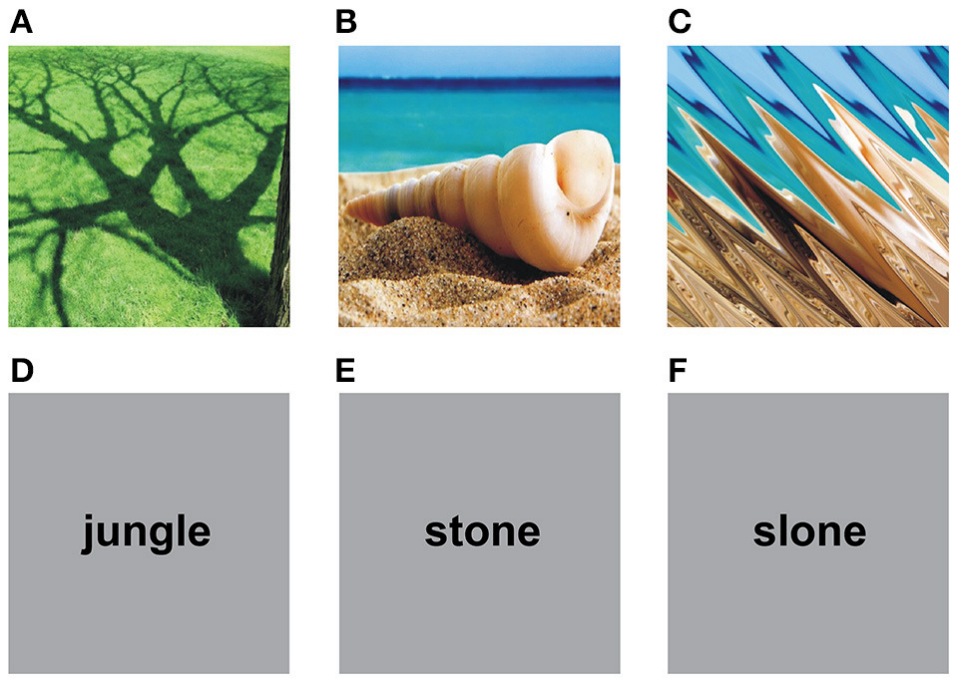
Examples of stimuli presented in the study. Upper row shows visual items while lower row shows verbal items. **(A)** A non-graspable object. **(B)** A graspable object. **(C)** A scrambled image. **(D)** A noun referring to a non-graspable object. **(E)** A noun referring to a graspable object. **(F)** A pseudo-word.

Thirty-six English nouns (see **Appendix 1**) referring to natural objects and 36 pseudowords as well as 36 digital color photos (see **Appendix 2**) depicting natural objects and 36 scrambled images were used as stimuli. Eighteen nouns referred to natural graspable objects (e.g., “leaf”) and 18 to natural non-graspable objects (e.g., “fog”). The pseudo-words were built by substituting one or two consonants or vowels in each noun (e.g., “leat” instead of “leaf”). With this procedure, pseudo-words contained orthographically and phonologically legal syllables for the English language. The photos depicted 18 graspable objects and 18 non-graspable objects. Figure 1 shows an example of each category. The scrambled images were built by applying an Adobe Illustrator distorting graphic filter (e.g., zigzag) to the photos depicting natural objects so to make them unrecognizable and then meaningless. All the photos and the scrambled images were 440 × 440 pixels.

The English nouns used as verbal stimuli and the nouns of the objects depicted in the photos were matched for word length [4.61, 4.72, 4.39, and 5.22 average letter number for graspable nouns, non-graspable nouns, graspable images, and non-graspable images, respectively; *F*_(3, 68)_ = 1.32, *p* = 0.27], syllable number [1.17, 1.33, 1.17, and 1.33 average syllable number; *F*_(3, 68)_ = 0.76, *p* = 0.52], and written lexical frequency [4.37, 4.68, 4.47, and 4.53 average number of occurrences per million in Google search engine; *F*_(3, 68)_ = 2.03, *p* = 0.12]. They were also matched for word imageability [i.e., how easily the word evokes a mental image of its referent; 5.36, 5.01, 5.23, and 5.17 average imageability score; *F*_(3, 68)_ = 1.53, *p* = 0.21] and familiarity [i.e., how often one encounters the word referent in natural environments; 4.79, 4.29, 4.28, and 4.32 average familiarity score; *F*_(3, 68)_ = 1.16, *p* = 0.33] as rated by 10 graduate and post-graduate student not involved in the experiment (7 females, mean age: 40.5 ± 12.9 years) using a seven-point scale (0: absent; 6 = extremely present). All the English nouns used as verbal stimuli and the nouns of the objects depicted in the photos had a reference level ranging between A1 and B1 except for 1 word of a B2 level on the CEFR scale. Whereas, the nouns of the objects depicted in the photos ranged from an A1 to a C1 level on the CEFR scale.

### Experimental design and procedure

The experiment was carried out in a sound-attenuated room, dimly illuminated by a halogen lamp directed toward the ceiling. Participants sat comfortably in front of a PC screen (HP 21.5′ LCD, 1,920 × 1,080 pixel resolution, and 60 Hz refresh rate). The eye-to-screen distance was about 57 cm.

**Figure 2:**
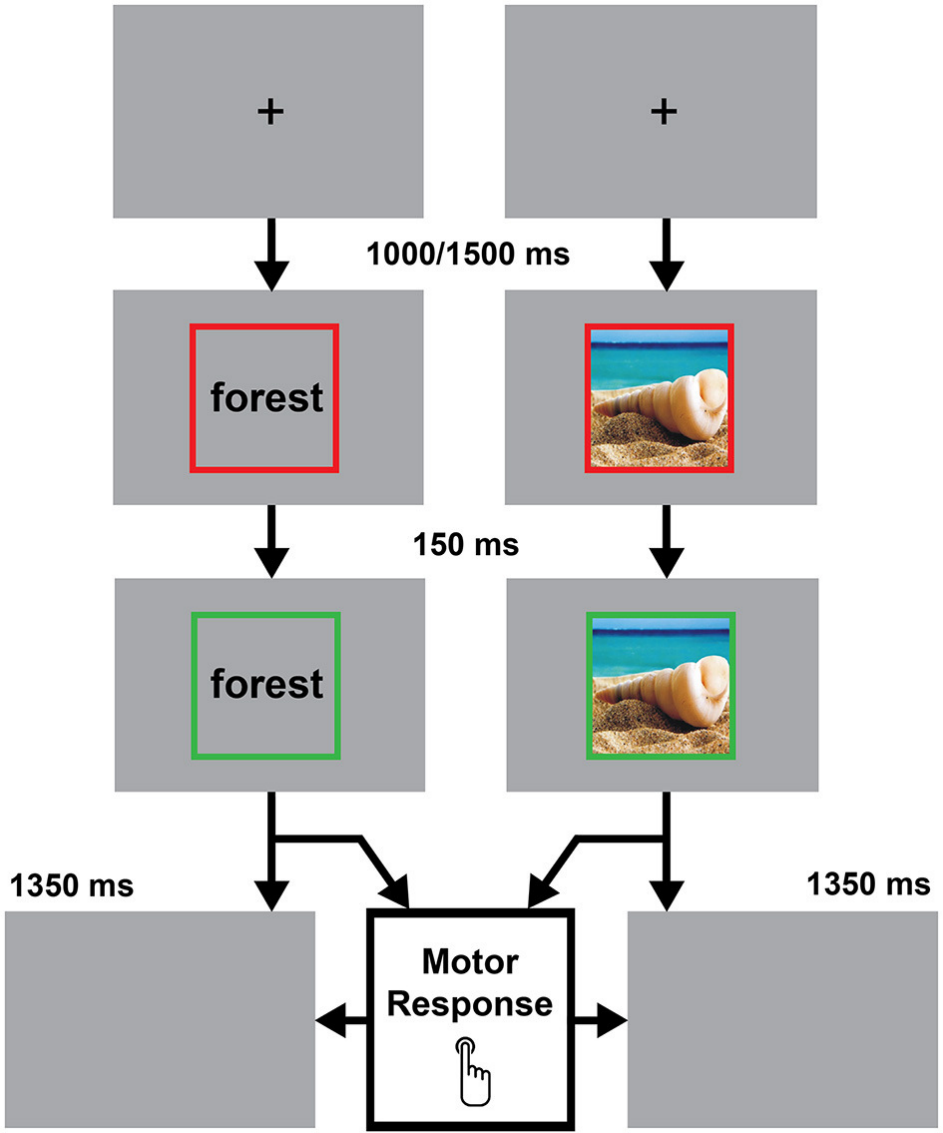
Schema of the experimental procedure used in the study. Each trial started with a fixation cross. The appearance of the green frame represented the go-signal. Stimuli remained visible until a motor response was given or the time limit had elapsed (1,350 ms).

Figure 2 shows the experimental procedure. Each trial started with a black fixation cross displayed at the center of a gray background. After a delay of 1,000–1,500 ms (in order to avoid response habituation), the fixation cross was replaced by a stimulus item, either a noun/pseudo-word or a photo/scrambled image. The verbal labels were written in black lowercase Courier New bold (font size = 24). Stimuli were centrally displayed and surrounded by a red (RGB coordinates = 255, 0, 0) 440 × 440 pixels frame (20 pixels-wide line). The red frame changed to green (RGB coordinates = 0, 255, 0) 150 ms after the stimulus onset. The color change of the frame was the “go” signal for the response. Participants were instructed to give a motor response, as fast and accurate as possible, by pressing a key on a computer keyboard centered on participants' body midline with their right index finger. They had to respond when the stimulus referred to a real object, and refrain from responding when it was meaningless (go-no go paradigm). Stimuli remained visible for 1,350 ms or until participant's response. Blanch A custom program developed in the MATLAB environment was used for stimulus presentation and response time collection.

The experiment consisted of 1 practice block and 1 experimental block. In the practice block, participants were presented with 32 stimuli (4 photos of graspable objects, 4 photos of non-graspable objects, 8 scrambled images, 4 nouns of graspable objects, 4 nouns of non-graspable objects, and 8 non-sense pseudowords) which were not used in the experimental block. During the practice block, participants received feedback (“ERROR”) after giving a wrong response (i.e., responding to a meaningless or refraining from responding to a real item), as well as for responses given prior to go signal presentation (“ANTICIPATION”), or later than 1.5 s (“YOU HAVE NOT ANSWERED”). In the experimental block, the 144 items selected as stimuli were randomly presented with the constraint that no more than three items of the same kind (verbal, visual) or referring to objects of the same category (graspable, non-graspable, meaningless) could be presented on consecutive trials. No feedback was given to participants. Thus, the experiment, which lasted about 20 min, consisted of 72 go trials (36 nouns of objects, 50% graspable and 50% non-graspable, plus 36 photographs of objects, 50% graspable, and 50% non-graspable) and 72 no-go trials (36 non-sense pseudowords plus 36 scrambled images), and 32 practice trials, for a total of 176 trials. To sum up, the experiment used a 2 × 2 repeated measures factorial design with Object Graspability (graspable, non-graspable) and Modality (verbal, visual) as the within-subjects variables.

## Results

Trials with errors were excluded without replacement. Errors were not further analyzed given they were extremely rare (<5%). Three participants were excluded from the analysis because their error rate exceeded 10%. Response times (RTs) below 130 ms or above 1,000 ms were omitted from the analysis. This cut-off was established so that no more than 0.5% of correct RTs were removed (Ulrich and Miller, 1994).

Median values of remaining RTs were calculated for each combination of Object Graspability (graspable and non-graspable) and Stimulus Type (photo and noun). These data entered a 2-way repeated measures analysis of variance (ANOVA) with Object Graspability and Stimulus Type as the within-subjects factors. Partial eta square values ($\eta_{\text{p}}^{2}$) are reported as an additional metric of effect size for all significant ANOVA contrasts.

The ANOVA revealed a main effect Object Graspability [*F*_(1, 22)_ = 11.87, *p* < 0.003, $\eta_{\text{p}}^{2}$ = 0.35], indicating that the participants gave slower responses to stimuli referring to graspable objects (387 ms ± 73) as compared to stimuli referring to non-graspable objects (371 ms ± 62). There was also a main effect of Stimulus Type [*F*_(1, 22)_ = 15.72, *p* < 0.001, $\eta_{\text{p}}^{2}$ = 0.42], reflecting slower responses to verbal stimuli than those to visual stimuli (395 ± 62 vs. 360 ms ± 70).

**Figure 3:**
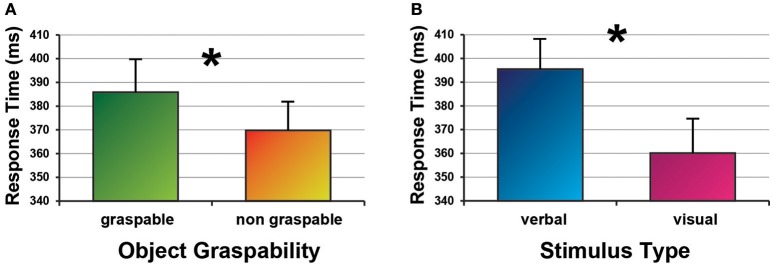
The main results of the experiment concerning the object graspability (graspable vs. non-graspable, **A**) and the modality of presentation (verbal vs. visual, **B**) are shown. Response times are expressed as means of medians. Asterisks mark significant effects.

Figure 3 shows the main results. Response times are expressed as means of medians.

## Discussion

During the processing of graspable objects and nouns presented in English, in the present study Italian participants, fluent speakers of English as L2, showed the same kind of modulation of motor responses as participants in a previous experiment (Marino et al., 2014), where the same kind of stimuli were presented in their L1. In details, participants gave slower reaction times during the processing of graspable items as compared to non-graspable ones, independent of presentation (noun or picture). We forward that, as for the L1, to solve the task participants relied on the motor representations of potential hand interactions with the object expressed by the noun or depicted in the photo. In this way, the motor system was engaged in two tasks at the same time, that is processing the presented stimuli and performing a motor response. Hence participants paid a cost as revealed by a slowing down of their motor responses. These results are relevant within the neuroscientific literature on L2. Ullman's differential hypothesis (Ullman, 2001) claims that L2 acquisition cannot depend on the same brain mechanisms that are used to process the native language. Coherently in earlier studies in bilingual aphasics the observation of selective recovery of one language was often interpreted as evidence for a different neural representation of L1 and L2 (Albert and Obler, 1978). More recently, several brain imaging studies have led to the notion that L1 and L2 are processed by the same neural structures (Perani and Abutalebi, 2005; Abutalebi, 2008). However, differential activations were found when the age of acquisition of L2 and the level of fluency are taken into account (Liu and Cao, 2016). When considering grammatical and syntactic processing, several studies (Sakai et al., 2004; Dodel et al., 2005; Ruschemeyer et al., 2005, 2006; Golestani et al., 2006; Indefrey, 2006; Jeong et al., 2007) showed stronger activations in L2 speakers within areas classically known as devoted to syntax (Grodzinsky and Friederici, 2006), including Broca's region and the adjacent left inferior frontal gyrus, left prefrontal cortex, basal ganglia and cerebellum. These studies included late bilinguals and their findings have been interpreted as due to a stronger effort in processing L2 as compared to L1. The very few studies that assessed early bilinguals showed that, as compared to late bilinguals, these individuals more strongly recruited left inferior frontal gyrus and prefrontal cortex (Wartenburger et al., 2003; Hernandez et al., 2007). As for semantics processing, studies in the field show that L2 is essentially processed through the same neural substrates underlying L1 processing, including anterior inferior frontal cortex and supramarginal gyrus. Differences related to L2 are found for low proficiency and/or less exposed bilinguals in terms of greater engagement of the left inferior frontal gyrus or selective engagement of prefrontal areas. It should be underlined that the age of L2 acquisition seems to have no major role in the semantics domain (Perani and Abutalebi, 2005; Indefrey, 2006). In other words L2 proficiency seems to be the main and only determinant in the semantics domain since late bilinguals with native like L2 proficiency activate the same identical areas for both languages. In the present study, we tested in a semantic task a rather homogenous group of students with a high competence in English as an L2. In keeping with the current literature, the present findings clearly show that motor responses to verbal stimuli presented in L2 are similarly modulated as in L1. This, in turn, suggests and supports at behavioral level the notion that the neural mechanisms (and possibly the neural substrates) underlying the processing of nouns in different languages are shared, and overlap with those necessary to process the corresponding objects, when presented pictorially. As a whole, experimental evidence supports the neural convergence hypothesis (Green, 2003), according to which the acquisition of an L2 relies on a specified language system devoted to L1 and claims that potential neural differences between L1 and L2 are overwhelmed as proficiency in L2 increases. It is worth stressing that at difference with the previous study (Marino et al., 2014) using L1 stimuli, in the present one we found a different role for modality of presentation. With L1 stimuli, motor responses to graspable objects were faster with nouns than with photos. In the present study, motor responses to graspable items were faster with photos than with verbal stimuli. We have no clear explanation for this finding. However, if it is true, as forwarded in our discussion that L2 verbal labels share the same neural representations as for L1, then it may be that while processing L2 verbal items participants also re-enacted the correspondent L1 verbal labels. This strategy, in turn, might have led to an additional cost when processing L2 items, as revealed behaviorally by a further slowing down of motor responses.

The present findings are also relevant within the embodiment literature. In a recent paper (Buccino et al., 2016), it has been suggested that meaning is strictly grounded in experience: the same neural mechanisms and neural substrates devoted to make sensory, motor and even emotional experience are also recruited and re-enacted when individuals have to attribute a meaning to language expressing those experiences. According to this proposal, the meaning of the word “flower,” for example, is not a particular flower or a bunch of flowers, and not even the stereotypical flower as a socially-defined entity that each speaker has to grasp in order to understand the meaning of that word. On the contrary, the word “flower” points at a cluster of flower-related real and concrete experiences that the speakers have made of that specific object called flower.

A recent proposal in the field of linguistics (Dor, 2015) seems to reach similar conclusions. This proposal defines a word as a “discrete instructor of imagination” whose basic function is to refer to and describe a set of personal experiences of the speakers. In other words, when communicating, the words have the primary role of expressing, on one side, a set of experiences that the utterer wants to focus on or convey, and of raising, on the other (or on the reader's side), an analogous set of personal experiences. Following this theoretical framework, the evidence that motor responses given to nouns presented in L2 were similar to those given for nouns in L1 (and for similar objects presented pictorially), strongly support the notion that, whatever the language used, at least for highly competent speakers, attributing a meaning to words implies re-enacting the neural substrates where experiences related to words are coded. Since we used graspable vs. non-graspable items, we found a specific modulation of motor responses common to the L1 and the L2.

In our opinion, these findings have implications in teaching and learning a second language. As discussed above, sensori-motor experience to which specific language elements refer appears central to language processing. If so, we believe that this notion is most relevant in second language learning and teaching: when a content has to be expressed and learned in a second language, it should refer to something which has already been experienced sensorially or motorically by the learner (Buccino and Mezzadri, 2015). This should lead language teachers (and learners) to adopt experience-based teaching methods whereby the content to be taught has to be targeted to the learner and revolve around the learner's experience. If experience does not support the language elements to be taught, the teacher should encourage the development of specific sensori-motor experiences which will then be verbally labeled. In other words, during the language teaching process the approach to any new language input should move from the (re)activation of pre-existing knowledge and experience. In keeping with this general statement, there is evidence that action may improve the acquisition of a foreign language or, in general, new words (Macedonia and von Kriegsten, 2012; Kronke et al., 2013) and may even be effective in the rehabilitation of language (Marangolo et al., 2010).

## Ethics statement

The study was conducted in accordance with the Declaration of Helsinki (1964) and the procedure recommended by the Italian Association of Psychology (AIP). Participants gave their informed consent before testing. The study was approved by the Ethical Committee of the University of Parma.

## Author contributions

GB contributed to plan the experiment, to collect, and analyze data and to write the manuscript; BM contributed to prepare stimuli and to collect and analyze data; CB contributed to prepare stimuli and to collect and analyze data; MM contributed to plan the experiment and write the manuscript.

### Conflict of interest statement

The authors declare that the research was conducted in the absence of any commercial or financial relationships that could be construed as a potential conflict of interest.

## Appendix 1

List of the English nouns used as verbal stimuli, their related pseudo-word, graspability of their referents, word frequency on Zipf scale (logarithmic frequency of occurrence) (van Heuven et al., 2014) word length (letter and syllable number), and reference level on CEFR scale verified with the English Vocabulary Profile [based on the Cambridge Learner Corpus (CLC), a multi-billion word corpus of spoken and written current English].

**Table:**

**English noun**	**Pseudo word**	**Ref grasp**	**Zipf-value**	**Letter/syllable**	**CEFR's level**
Ankle	Antle	Yes	3.94	5/1	B1
Beard	Beaft	Yes	3.98	5/1	A1
Bone	Bose	Yes	4.51	4/1	B1
Diamond	Diafond	Yes	4.39	7/2	B2
Dust	Dunt	Yes	4.39	4/1	B1
Ear	Eaz	Yes	4.41	3/1	A1
Flower	Floter	Yes	4.50	6/2	A1
Fur	Fup	Yes	4.03	3/1	B1
Garlic	Gatlic	Yes	4.45	6/2	A2
Grass	Grast	Yes	4.58	5/1	A1
Herbs	Hergs	Yes	4.02	5/1	B1
Knee	Snee	Yes	4.26	4/1	B1
Leaf	Leat	Yes	4.29	4/1	B1
Plant	Plaft	Yes	4.81	5/1	A1
Salt	Sart	Yes	4.69	4/1	A1
Sand	Sast	Yes	4.51	4/1	B1
Stone	Slone	Yes	4.84	5/1	B1
Wool	Woop	Yes	4.11	4/1	A2
Air	Aiz	No	5.22	3/1	A2
Bay	Zay	No	4.55	3/1	B1
Cliff	Tliff	No	4.37	5/1	B1
Fog	Fot	No	4.08	3/1	A2
Forest	Fodest	No	4.67	6/1	A2
Heat	Heas	No	4.76	4/1	B1
Hill	Hiln	No	4.71	4/1	A2
Island	Istand	No	4.96	6/2	A2
Jungle	Rungle	No	4.38	6/1	B1
Lake	Lare	No	4.49	4/1	A2
Moon	Moof	No	4.74	4/1	A2
Rain	Raim	No	5.08	4/1	A1
Sea	Xea	No	5.20	3/1	A1
Space	Spale	No	5.29	5/1	B1
Thunder	Thunser	No	3.93	7/2	B1
Universe	Unigerse	No	4.37	8/3	B1
Valley	Vatley	No	4.47	6/2	B1
Wind	Winz	No	5.00	4/1	A1

## Appendix 2

List of the nouns of the objects depicted in the photos used as visual stimuli, their word frequency on Zipf scale (logarithmic frequency of occurrence), word length (letter and syllable number), and reference level on CEFR scale verified with the English Vocabulary Profile [based on the Cambridge Learner Corpus (CLC), a multi-billion word corpus of spoken and written current English].

**Table:**

**English noun**	**Ref grasp**	**Zipf-value**	**Letter/Syllable**	**CEFR's level**
Branch	Yes	4.10	6/1	B1
Bulb	Yes	3.68	4/1	B2
Coal	Yes	4.32	4/1	C1
Daisy	Yes	4.61	5/1	NA
Egg	Yes	4.80	3/1	A1
Gold	Yes	5.10	4/1	A2
Hair	Yes	5.03	4/2	A1
Honey	Yes	4.60	5/2	A2
Ice	Yes	4.98	3/1	A2
Net	Yes	4.53	3/1	B1
Nut	Yes	3.99	3/1	B2
Parsley	Yes	3.98	7/2	NA
Pepper	Yes	4.38	6/2	B1
Shell	Yes	4.39	5/1	B2
Snow	Yes	4.79	4/1	A1
Tail	Yes	4.45	4/1	B2
Trunk	Yes	3.93	5/1	B2
Wood	Yes	4.78	4/1	A2
Beach	No	4.72	5/1	A1
Cavern	No	3.08	6/2	NA
Cell	No	4.22	4/1	B2
Cloud	No	4.77	5/1	A2
Coast	No	4.86	5/1	B1
Earth	No	5.00	5/1	B1
Field	No	4.92	5/1	A2
Landscape	No	4.62	9/2	B1
Mountain	No	4.64	8/2	A2
Ocean	No	4.49	5/2	B1
Planet	No	4.66	6/2	B1
Pond	No	4.20	4/1	B2
Shadow	No	4.49	6/2	B1
Shore	No	4.12	5/1	B1
Sky	No	4.79	3/1	A2
Smoke	No	4.53	5/1	B1
Storm	No	4.46	5/1	A2

## References

[B1] Abutalebi J. (2008). Neural aspects of second language representation and language control. Acta Psychol. 128, 466–478. 10.1016/j.actpsy.2008.03.014 18479667

[B2] Albert M. Obler L. (1978). The Bilingual Brain: Neuropsychological and Neurolinguistic Aspects of Bilingualism. New York, NY: Academic Press.

[B3] Barsalou L. W. (1999). Perceptual symbol systems. Behav. Brain Sci. 22, 577–609; discussion 610–560. 10.1017/S0140525X99002149 11301525

[B4] Binkofski F. Buccino G. Posse S. Seitz R. J. Rizzolatti G. Freund H. (1999). A fronto-parietal circuit for object manipulation in man: evidence from an fMRI-study. Eur. J. Neurosci. 11, 3276–3286. 10.1046/j.1460-9568.1999.00753.x 10510191

[B5] Bub D. N. Masson M. E. Cree G. S. (2008). Evocation of functional and volumetric gestural knowledge by objects and words. Cognition 106, 27–58. 10.1016/j.cognition.2006.12.010 17239839

[B6] Buccino G. Mezzadri M. (2015). Embodied language and the process of language learning and teaching, in Emotion in Language: Theory – Research – Application, ed Lüdtke U. M. (Amsterdam: John Benjamins Publishing Company), 191–208.

[B7] Buccino G. Colage I. Gobbi N. Bonaccorso G. (2016). Grounding meaning in experience: a broad perspective on embodied language. Neurosci. Biobehav. Rev. 69, 69–78. 10.1016/j.neubiorev.2016.07.033 27477443

[B8] Buccino G. Sato M. Cattaneo L. Roda F. Riggio L. (2009). Broken affordances, broken objects: a TMS study. Neuropsychologia 47, 3074–3078. 10.1016/j.neuropsychologia.2009.07.003 19615389

[B9] Cattaneo Z. Devlin J. T. Salvini F. Vecchi T. Silvanto J. (2010). The causal role of category-specific neuronal representations in the left ventral premotor cortex (PMv) in semantic processing. Neuroimage 49, 2728–2734. 10.1016/j.neuroimage.2009.10.048 19853046

[B10] Chao L. L. Martin A. (2000). Representation of manipulable man-made objects in the dorsal stream. Neuroimage 12, 478–484. 10.1006/nimg.2000.0635 10988041

[B11] Chersi F. Thill S. Ziemke T. Borghi A. M. (2010). Sentence processing: linking language to motor chains. Front. Neurorobot. 4:4. 10.3389/fnbot.2010.00004 20725506 PMC2901116

[B12] Desai R. H. Choi W. Lai V. T. Henderson J. M. (2016). Toward semantics in the wild: activation to manipulable nouns in naturalistic reading. J. Neurosci. 36, 4050–4055. 10.1523/JNEUROSCI.1480-15.2016 27053211 PMC4821914

[B13] Dodel S. Golestani N. Pallier C. Elkouby V. Le Bihan D. Poline J. B. (2005). Condition-dependent functional connectivity: syntax networks in bilinguals. Philos. Trans. R. Soc. Lond. B. Biol. Sci. 360, 921–935. 10.1098/rstb.2005.1653 16087437 PMC1854936

[B14] Dor D. (2015). The Instruction of Imagination. Language as a Social Communication Technology. Oxford: Oxford University Press; Foundations of Human Interaction.

[B15] Gallese V. Lakoff G. (2005). The Brain's concepts: the role of the Sensory-motor system in conceptual knowledge. Cogn. Neuropsychol. 22, 455–479. 10.1080/02643290442000310 21038261

[B16] Ganis G. Kutas M. Sereno M. I. (1996). The search for “common sense”: an electrophysiological study of the comprehension of words and pictures in reading. J. Cogn. Neurosci. 8, 89–106. 10.1162/jocn.1996.8.2.89 23971417

[B17] Garcia A. M. Ibanez A. (2016). A touch with words: dynamic synergies between manual actions and language. Neurosci. Biobehav. Rev. 68, 59–95. 10.1016/j.neubiorev.2016.04.022 27189784

[B18] Glenberg A. M. (1997). What memory is for. Behav. Brain Sci. 20, 1–19; discussion 19–55. 10.1017/S0140525X97000010 10096994

[B19] Glover S. Rosenbaum D. A. Graham J. Dixon P. (2004). Grasping the meaning of words. Exp. Brain Res. 154, 103–108. 10.1007/s00221-00-1659-2 14578997

[B20] Golestani N. Alario F. X. Meriaux S. Le Bihan D. Dehaene S. Pallier C. (2006). Syntax production in bilinguals. Neuropsychologia 44, 1029–1040. 10.1016/j.neuropsychologia.2005.11.009 16427099

[B21] Gough P. M. Campione G. C. Buccino G. (2013). Fine tuned modulation of the motor system by adjectives expressing positive and negative properties. Brain Lang. 125, 54–59. 10.1016/j.bandl.2013.01.012 23454074

[B22] Gough P. M. Riggio L. Chersi F. Sato M. Fogassi L. Buccino G. (2012). Nouns referring to tools and natural objects differentially modulate the motor system. Neuropsychologia 50, 19–25. 10.1016/j.neuropsychologia.2011.10.017 22044649

[B23] Green D. W. (2003). Neural basis of lexicon and grammar in L2 acquisition. The convergence hypothesis, in The Lexicon Syntax Interface in Second Language Acquisition, eds Van Hout R. Hulk A. Kuiken F. Towell R. J. (Amsterdam; Philadelphia, PA: John Benjamins Publishing Company), 197–218.

[B24] Grezes J. Armony J. L. Rowe J. Passingham R. E. (2003a). Activations related to “mirror” and “canonical” neurones in the human brain: an fMRI study. Neuroimage 18, 928–937. 10.1016/S1053-8119(03)00042-9 12725768

[B25] Grezes J. Tucker M. Armony J. Ellis R. Passingham R. E. (2003b). Objects automatically potentiate action: an fMRI study of implicit processing. Eur. J. Neurosci. 17, 2735–2740. 10.1046/j.1460-9568.2003.02695.x 12823480

[B26] Grodzinsky Y. Friederici A. D. (2006). Neuroimaging of syntax and syntactic processing. Curr. Opin. Neurobiol. 16, 240–246. 10.1016/j.conb.2006.03.007 16563739

[B27] Hernandez A. E. Hofmann J. Kotz S. A. (2007). Age of acquisition modulates neural activity for both regular and irregular syntactic functions. Neuroimage 36, 912–923. 10.1016/j.neuroimage.2007.02.055 17490895 PMC1995424

[B28] Indefrey P. (2006). A meta-analysis of hemodynamic studies on first and second language processing: which suggested differences can we trust and what do they mean? Lang. Learn. 56, 279–304. 10.1111/j.1467-9922.2006.00365.x

[B29] Jeannerod M. Arbib M. A. Rizzolatti G. Sakata H. (1995). Grasping objects: the cortical mechanisms of visuomotor transformation. Trends Neurosci. 18, 314–320. 10.1016/0166-2236(95)93921-J 7571012

[B30] Jeong H. Sugiura M. Sassa Y. Haji T. Usui N. Taira M.. (2007). Effect of syntactic similarity on cortical activation during second language processing: a comparison of English and Japanese among native Korean trilinguals. Hum. Brain Mapp. 28, 194–204. 10.1002/hbm.20269 16767768 PMC6871317

[B31] Jirak D. Menz M. M. Buccino G. Borghi A. M. Binkofski F. (2010). Grasping language–a short story on embodiment. Conscious. Cogn. 19, 711–720. 10.1016/j.concog.2010.06.020 20739194

[B32] Kronke K. M. Mueller K. Friederici A. D. Obrig H. (2013). Learning by doing? The effect of gestures on implicit retrieval of newly acquired words. Cortex 49, 2553–2568. 10.1016/j.cortex.2012.11.016 23357203

[B33] Lindemann O. Stenneken P. van Schie H. T. Bekkering H. (2006). Semantic activation in action planning. J. Exp. Psychol. Hum. Percept. Perform. 32, 633–643. 10.1037/0096-1523.32.3.633 16822129

[B34] Liu H. Cao F. (2016). L1 and L2 processing in the bilingual brain: a meta-analysis of neuroimaging studies. Brain Lang. 159, 60–73. 10.1016/j.bandl.2016.05.013 27295606

[B35] Macedonia M. von Kriegsten K. (2012). Gestures enhance foreign language learning. Biolinguistics 6, 393–416.

[B36] Makris S. Hadar A. A. Yarrow K. (2011). Viewing objects and planning actions: on the potentiation of grasping behaviours by visual objects. Brain Cogn. 77, 257–264. 10.1016/j.bandc.2011.08.002 21903319

[B37] Marangolo P. Bonifazi S. Tomaiuolo F. Craighero L. Coccia M. Altoe G.. (2010). Improving language without words: first evidence from aphasia. Neuropsychologia 48, 3824–3833. 10.1016/j.neuropsychologia.2010.09.025 20887740

[B38] Marino B. F. Gough P. M. Gallese V. Riggio L. Buccino G. (2013). How the motor system handles nouns: a behavioral study. Psychol. Res. 77, 64–73. 10.1007/s00426-011-0371-2 21879354

[B39] Marino B. F. Sirianni M. Volta R. D. Magliocco F. Silipo F. Quattrone A.. (2014). Viewing photos and reading nouns of natural graspable objects similarly modulate motor responses. Front. Hum. Neurosci. 8:968. 10.3389/fnhum.2014.00968 25538596 PMC4255516

[B40] Myung J. Y. Blumstein S. E. Sedivy J. C. (2006). Playing on the typewriter, typing on the piano: manipulation knowledge of objects. Cognition 98, 223–243. 10.1016/j.cognition.2004.11.010 16399263

[B41] Perani D. Abutalebi J. (2005). The neural basis of first and second language processing. Curr. Opin. Neurobiol. 15, 202–206. 10.1016/j.conb.2005.03.007 15831403

[B42] Pulvermüller F. (2001). Brain reflections of words and their meaning. Trends Cogn. Sci. 5, 517–524. 10.1016/S1364-6613(00)01803-9 11728909

[B43] Pulvermüller F. Shtyrov Y. Hauk O. (2009). Understanding in an instant: neurophysiological evidence for mechanistic language circuits in the brain. Brain Lang. 110, 81–94. 10.1016/j.bandl.2008.12.001 19664815 PMC2734884

[B44] Ruschemeyer S. A. Fiebach C. J. Kempe V. Friederici A. D. (2005). Processing lexical semantic and syntactic information in first and second language: fMRI evidence from German and Russian. Hum. Brain Mapp. 25, 266–286. 10.1002/hbm.20098 15849713 PMC6871675

[B45] Ruschemeyer S. A. Zysset S. Friederici A. D. (2006). Native and non-native reading of sentences: an fMRI experiment. Neuroimage 31, 354–365. 10.1016/j.neuroimage.2005.11.047 16427323

[B46] Sakai K. L. Miura K. Narafu N. Muraishi Y. (2004). Correlated functional changes of the prefrontal cortex in twins induced by classroom education of second language. Cereb. Cortex 14, 1233–1239. 10.1093/cercor/bhh084 15142962

[B47] Tucker M. Ellis R. (2004). Action priming by briefly presented objects. Acta Psychol. 116, 185–203. 10.1016/j.actpsy.2004.01.004 15158182

[B48] Ullman T. L. (2001). The neural basis of lexicon and grammar in first and second language: the declarative/procedural model. Bilingual. Lang. Cogn. 4, 105–122. 10.1017/S1366728901000220

[B49] Ulrich R. Miller J. (1994). Effects of truncation on reaction time analysis. J. Exp. Psychol. Gen. 123, 34–80. 10.1037/0096-3445.123.1.34 8138779

[B50] van Heuven W. J. Mandera P. Keuleers E. Brysbaert M. (2014). SUBTLEX-UK: a new and improved word frequency database for British English. Q. J. Exp. Psychol. (Hove). 67, 1176–1190. 10.1080/17470218.2013.850521 24417251

[B51] Vandenberghe R. Price C. Wise R. Josephs O. Frackowiak R. S. (1996). Functional anatomy of a common semantic system for words and pictures. Nature 383, 254–256. 10.1038/383254a0 8805700

[B52] Wartenburger I. Heekeren H. R. Abutalebi J. Cappa S. F. Villringer A. Perani D. (2003). Early setting of grammatical processing in the bilingual brain. Neuron 37, 159–170. 10.1016/S0896-6273(02)01150-9 12526781

[B53] Zhang Z. Sun Y. Humphreys G. W. (2016). Perceiving object affordances through visual and linguistic pathways: a comparative study. Sci. Rep. 6:26806. 10.1038/srep26806 27222369 PMC4879702

[B54] Zwaan R. A. Taylor L. J. (2006). Seeing, acting, understanding: motor resonance in language comprehension. J. Exp. Psychol. Gen. 135, 1–11. 10.1037/0096-3445.135.1.1 16478313

